# Plant Secondary Metabolites Produced in Response to Abiotic Stresses Has Potential Application in Pharmaceutical Product Development

**DOI:** 10.3390/molecules27010313

**Published:** 2022-01-05

**Authors:** Karma Yeshi, Darren Crayn, Edita Ritmejerytė, Phurpa Wangchuk

**Affiliations:** 1 Centre for Molecular Therapeutics, Australian Institute of Tropical Health and Medicine, James Cook University, Building E4, McGregor Rd, Smithfield, Cairns, QLD 4878, Australia; edita.ritmejeryte@jcu.edu.au (E.R.); phurpa.wangchuk@jcu.edu.au (P.W.); 2 Australian Tropical Herbarium, James Cook University, Building E1, McGregor Rd, Smithfield, Cairns, QLD 4878, Australia; darren.crayn@jcu.edu.au

**Keywords:** secondary metabolites, climate change, drug discovery, abiotic stress

## Abstract

Plant secondary metabolites (PSMs) are vital for human health and constitute the skeletal framework of many pharmaceutical drugs. Indeed, more than 25% of the existing drugs belong to PSMs. One of the continuing challenges for drug discovery and pharmaceutical industries is gaining access to natural products, including medicinal plants. This bottleneck is heightened for endangered species prohibited for large sample collection, even if they show biological hits. While cultivating the pharmaceutically interesting plant species may be a solution, it is not always possible to grow the organism outside its natural habitat. Plants affected by abiotic stress present a potential alternative source for drug discovery. In order to overcome abiotic environmental stressors, plants may mount a defense response by producing a diversity of PSMs to avoid cells and tissue damage. Plants either synthesize new chemicals or increase the concentration (in most instances) of existing chemicals, including the prominent bioactive lead compounds morphine, camptothecin, catharanthine, epicatechin-3-gallate (EGCG), quercetin, resveratrol, and kaempferol. Most PSMs produced under various abiotic stress conditions are plant defense chemicals and are functionally anti-inflammatory and antioxidative. The major PSM groups are terpenoids, followed by alkaloids and phenolic compounds. We have searched the literature on plants affected by abiotic stress (primarily studied in the simulated growth conditions) and their PSMs (including pharmacological activities) from PubMed, Scopus, MEDLINE Ovid, Google Scholar, Databases, and journal websites. We used search keywords: “stress-affected plants,” “plant secondary metabolites, “abiotic stress,” “climatic influence,” “pharmacological activities,” “bioactive compounds,” “drug discovery,” and “medicinal plants” and retrieved published literature between 1973 to 2021. This review provides an overview of variation in bioactive phytochemical production in plants under various abiotic stress and their potential in the biodiscovery of therapeutic drugs. We excluded studies on the effects of biotic stress on PSMs.

## 1. Introduction

Plant secondary metabolites (PSMs) are small molecules with diverse chemical structures and biological activities. Unlike primary metabolites, which are the main drivers of essential life functions, including cell formation, PSMs are neither necessary for primary life functions nor possess high-energy bonds [1]. However, PSMs play essential secondary physiological and biochemical functions that ensure plant fitness and survival, particularly concerning their interactions with the environment and coping with biotic and abiotic stress [1]. These factors, especially abiotic stressors (nutrient deficiencies, seasons, salinity, wounding, drought, light, UV radiation, temperature, greenhouse gases, and climate changes), cause significant perturbations in chemotypes and levels of PSMs production. For example, plants produce more terpenoids when exposed to high temperatures [2], and UV-B (280–315 nm) radiation induces tree foliage to produce more phenolic acids and flavonoids as protective pigments [3,4]. Phenolics and flavonoids are well-known for their antioxidative and anti-inflammatory properties [5,6,7]. Similarly, the production of antioxidative compounds such as glutathione, g-aminobutyric acid (GABA), terpenoids, and volatile organic compounds (VOCs) increases under elevated O_3_ [8].

PSMs are vital for human health and form many pharmaceutical drugs’ backbone. Indeed, more than 25% of the existing drugs belong to PSMs [9]. The most popular PSMs-derived drugs are morphine (isolated from *Papaver somniferum*), digitoxin (isolated from *Digitalis purpurea*), taxol (isolated from *Taxus baccata*), artemisinin (isolated from *Artemisia annua*) and quinine (isolated from *Cinchona officinalis*), vinblastine and vincristine (isolated from *Catharanthus roseus*); and aspirin (first isolated as salicylic acid from *Filipendula ulmaria*). Since plants exposed to various abiotic stress conditions produce many PSMs in higher concentrations as their coping mechanism [10,11,12], it presents opportunities for natural product researchers and pharmaceutical companies to explore the biochemical responses of plants to climatic stress for developing many novel therapeutics. However, there is no comprehensive literature review examining the scope of plants affected by abiotic stresses for drug discovery.

Therefore, this scoping review examines recent advances related to PSMs in plants affected by abiotic stress/or abiotic growth factors, their roles as protective phytochemicals, and their potential for novel drug lead compounds. Although primary metabolites such as carbohydrates [13,14] and peptides [15,16] are also known to play roles in the plant’s defense response, our review focuses on selected classes of PSMs, including flavonoids, terpenoids, alkaloids, saponins, tannins, and cyanogenic glycosides. We have collected published information on plants affected by abiotic stresses (primarily studied in the simulated growth conditions) and their PSMs (including pharmacological activities) from PubMed, Scopus, MEDLINE Ovid, Google Scholar, Databases, and journal websites using the following keywords: “stress-affected plants,” “plant secondary metabolites,” “bioactive compounds,” “abiotic stress,” “climatic influence,” “pharmacological activities,” “drug discovery,” and “medicinal plants.” We have retrieved published literature between 1973 to 2021 (only related to PSMs produced under ex situ growth conditions), analysed the content, and presented the information in the form of figures and tables. The chemical structures were drawn by using ChewDraw Professional software, and each structure was cross-checked for their correctness using ChemSpider and HMDB databases. We excluded studies on the effects of biotic stress on PSMs.

## 2. Plant Secondary Metabolites and Their Biological Roles

Generally, all plants produce secondary metabolites for defense, attraction, communication, and mediating stress [17]. For example, plants produce VOCs as defense molecules, and they are known to function as antimicrobial and insect repellent agents [18]. More than 200,000 PSMs have been identified [19], and with more than 391,000 plant species known worldwide [20], there is space for more discoveries. Some PSMs are specific to certain related plant taxa [21], and their concentrations can vary between populations and individual plants with plant ontogeny and tissue type [22,23]. These PSM variations can be due to genetic variability, but their concentrations are affected by environmental abiotic factors (growth conditions) such as those expected to intensify with climate change (e.g., heat stress, drought, UV radiation, and O_3_) [24], and herbivore and pathogen attacks [25,26]. Based on a biosynthetic pathway and chemical structure, PSMs have broadly been categorized into three major groups: (i) terpenoids (plant volatiles, sterols, carotenoids, saponins, and glycosides), (ii) phenolic compounds (flavonoids, phenolic acids, lignin, lignans, coumarins, stilbenes, and tannins), and (iii) nitrogen-containing compounds (alkaloids, glucosinolates, and cyanogenic glycosides) [27,28,29,30].

### 2.1. Terpenoids

**Figure 1:**
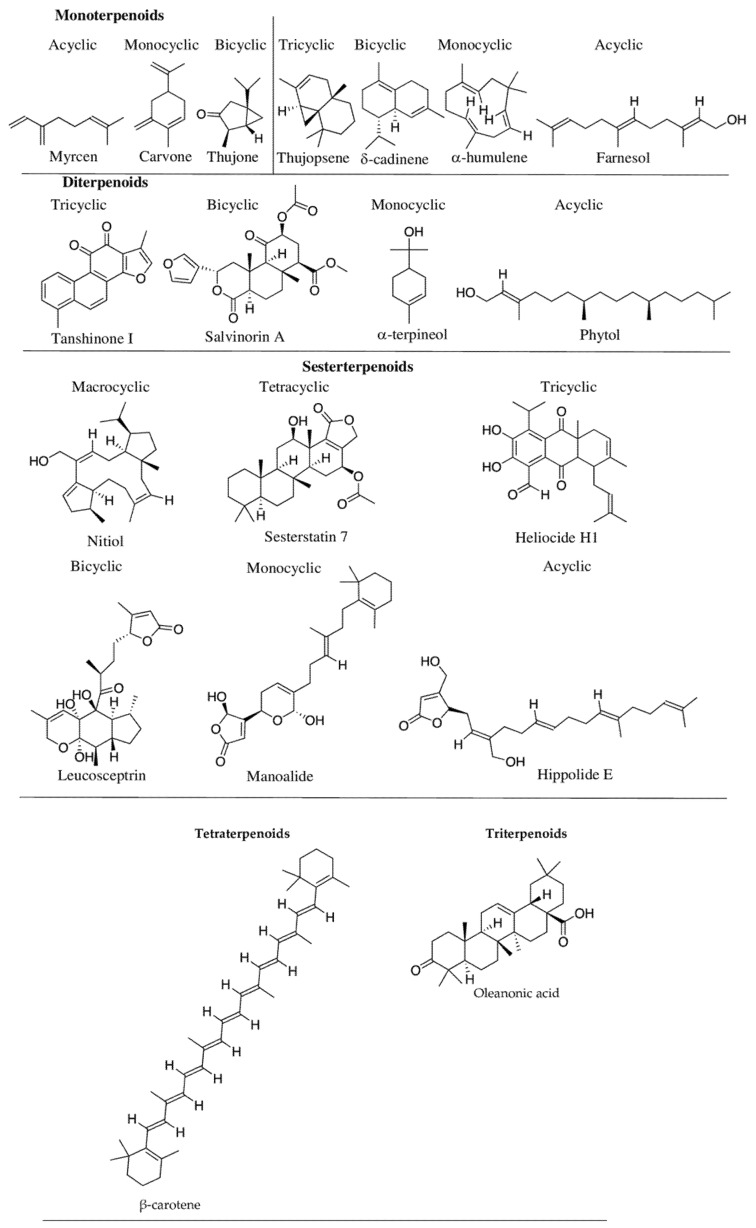
Representative examples of terpenoid plant secondary metabolites.

Terpenoids or isoprenoids are one of the most structurally diverse naturally occurring PSMs, with the main skeleton consisting of five-carbon isopentyl units, called 2-methyl-1,3-butadiene, or isoprene. Terpenes contain only isoprene units, while terpenoids have additional functional groups, such as ketone or heterocyclic and hydroxyl rings. Based on structural construction, terpenoids can be considered as two types, aliphatic (e.g., geraniol) and cyclic (e.g., limonene) terpenoids. Since terpenoids contain many isoprene units, they are divided into various groups, as described below (Figure 1):

- Monoterpenoids—two isoprene units (C-10 carbon atoms,)—e.g., linalool;
- Sesquiterpenoids—three isoprene units (C-15 carbon atoms)—e.g., β-caryophyllene;
- Diterpenoids—four isoprene units (C-20 carbon atoms)—e.g., abietic acid;
- Sesterterpenoids—five isoprene units (C-25 carbon atoms)—e.g., ophiobolin A;
- Triterpenoids—six isoprene units (C-30 carbon atoms)—e.g., ganoderic acid;
- Tetraterpenoids—eight isoprene units (C-40 carbon atoms)—e.g., α-carotene;
- Polyterpenoids—more than eight isoprene units (>C-40 carbon atoms)—e.g., trans-1,4-polyisoprene.

Terpenoids are formed from the mevalonate pathway inside cytosol or the 2-C-methyl-D-erythritol-4-phosphate (MEP) pathway inside the plastid [31]. The biosynthetic precursors of terpenoids include geranyl diphosphate (GPP) for monoterpenes; farnesyl diphosphate (FPP) for sesquiterpenes; and geranylgeranyl diphosphate (GGPP) for diterpenes [32]. An in-depth discussion on terpenoids’ biosynthetic pathway and structural diversity is covered by Aharoni et al. (2005) and Song et al. (2014) in their review [31,32]. More than 30,000 terpenes have been reported to date [33]. They are mostly phytohormones (e.g., gibberellins), photosynthetic pigments (e.g., phytol, carotenoids such as α-carotene and β-carotene), and carriers (e.g., ubiquinone, plastoquinone) in the electron chain transport systems [34,35]. The role of terpenoids is to protect plants directly (e.g., releasing phytoalexins after pathogen attacks) or indirectly by producing mixtures of volatile organic compounds (VOCs) to attract carnivores of their herbivores [36]. Phytoalexins are antimicrobial compounds produced after microbes’ challenge plants, and it is reviewed in-depth by González-Lamothe et al. [37]. VOCs include terpenoids (isoprene or hemiterpenoids and monoterpenoids), alkanes, alkenes, carbonyls, alcohols, esters, ethers, and acids [18]. VOCs are involved in plant-plant or plant-insect interactions, but some terpenoids act as lipid-soluble antioxidants inducing resistance to stress [38].

Several terpenoids have shown defensive roles against biotic and abiotic stresses in plants. Dahham et al. (2015) [39] and Porres-Martínez et al. (2016) [40] reported antioxidant activities of terpenes (sesquiterpene b-caryophyllene, and monoterpenes 1,8-cineole and α-pinene, respectively), suggesting their function in overcoming abiotic-induced oxidative stress. Terpenoids are also reported to protect plants from photodamage and oxidative stress by supporting photorespiration [41]. Carotenoids are the best-known terpenoids involved in photoprotection [42]. Other examples of defensive terpenes in plants are triterpene glycosides (or saponins) such as α-tomatine in the fruits and leaves of tomatoes [43] and avenacin in oat (*Avena sativa*) roots [44]. Avenacin and α-tomatine are important pre-formed antimicrobial compounds, commonly referred to as phytoanticipins, and they have defensive roles against microbial attacks [37]. Saponins are another group of compounds under terpenoids with triterpenes or steroidal aglycones linked to one or more sugar chains [45], but some saponins such as steroidal glycoalkaloids have a nitrogen atom in their aglycone chemical structure [45,46]. Similarl to many other PSMs, the amount and distribution of saponins in plants are influenced by season, biotic and abiotic stresses, and plant developmental stage. For example, maximum saponin production in *Phytolacca dodecandra* L’Hér. [47] and *Dioscorea pseudojaponica* Yamamoto [48] occurs during fruit and tuber development to prevent fruit loss and enable seed maturation. Under stress conditions, saponin levels in plants increase through jasmonate and salicylate signaling pathways [45].

### 2.2. Phenolic Compounds

More than 8000 phenolic compounds are reported from plants, of which half of them are flavonoids (approximately 4000–4500 compounds) such as aglycone, glycosides, and methylated derivatives [6,49]. Phenolics exhibit diverse structures from single aromatic rings (e.g., in phloroglucinol, gentisic acid, ferulic acid, caffeic acid, and vanillin) to complex polymeric structures such as in lignins (e.g., coniferyl alcohol), coumarins (e.g., scopoletin), phenolic quinones (e.g., juglone), tannins (e.g., ellagic acid), and flavonoids [50,51]. Among phenolic group compounds, flavonoids are the most abundant, and stilbenes and lignans are less common.

**Figure 2:**
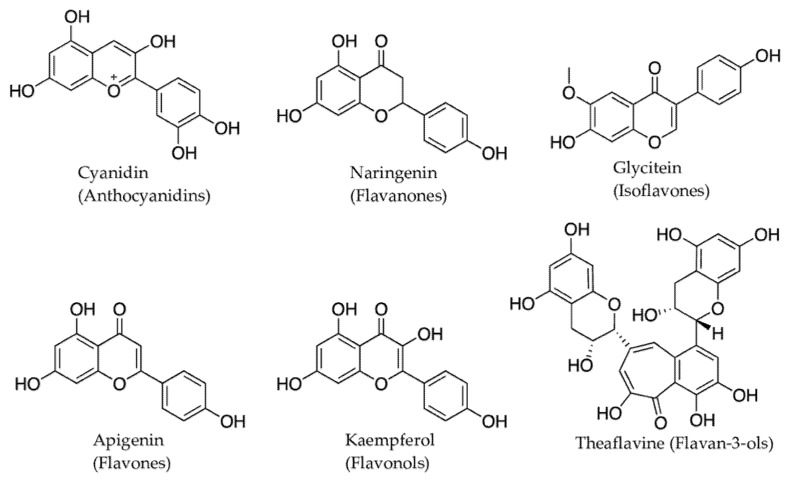
Representative examples of different subgroups of flavonoids: a major phenolic group of secondary metabolites.

Flavonoids are a diverse secondary metabolite group with a wide array of functions, including protection against stress. Flavonoids comprise seven sub-groups (Figure 2) (flavones, flavonols, flavanones, isoflavonoids, flavan-3-ols or catechins, and anthocyanins) [52,53] based on the C-ring carbon to which B-ring is attached, and also based on the degree of oxidation and unsaturation of their C-ring [53]. Flavones contain a double bond between positions 2 and 3 and a ketone functional group in position 4 of the C-ring. In comparison, flavonols have a hydroxy group at position 3 of the C-ring and are sometimes glycosylated. Unlike flavones, flavonones are saturated with a double bond between positions 2 and 3 of the C-ring. Flavan-3-ols have a hydroxyl group in position 3 of the C-ring, but there is no double bond between positions 2 and 3 [53]. Although the metabolic role of phenolics is not well-defined, their protective functions in plants are attributed to their ability to scavenge free radicals and filter harmful UV radiations [54,55]. Ferulic acid, caffeic acid, and *p*-coumaric acid (hydroxycinnamic acid derivatives) are some of the best-known UV-B attenuators in plants [56]. Flavonoids help plants adjust to extreme heat and cold [57] through increasing accumulation. When Schulz et al. (2016) [58] analyzed the expression of flavonoids in 20 mutants of two different *Arabidopsis thaliana accessions* (Col-0 and Ler) in response to freezing and cold acclimation (14 days at 4 °C), 19 mutants, which are gene-knock outs, did not exhibit flavonoid biosynthesis, with an exception to pap1-D mutant. A similar observation of increasing concentrations in flavonoids (anthocyanins and flavonols) was also reported by Pastore et al. (2017) [59] in grapevine berries, but tannins did not show any changes. The role of flavonoids in UV protection is also supported by Bieza and Lois’ work [60], in which they have isolated an *Arabidopsis* mutant tolerant to high levels of UV-B radiations. Such protective flavonoids are reported more in plants thriving in colder climates at higher elevations and semi-arid environments [61]. Flavonoid-based plant pigments, such as anthocyanins synthesized in the last step of the flavonoid biosynthesis pathway under UV stress upon acylation, can absorb UV radiation and scavenge ROS [62,63]. If not kept under control, ROS can cause direct damage to plants through the oxidation of essential biomolecules, leading to the accumulation of more ROS and ultimately programmed cell death [64].

Flavonoids such as quercetin can chelate transition metals (for example, Fe), consequently inhibiting Fenton reaction (conversion of H_2_O_2_ to toxic OH^•^ radical), thereby creating a robust antioxidative environment in the plants [65]. Phenolics are also known to play a strategic role in reproduction as frugivore attractants that promote seed dispersal (e.g., anthocyanidins and anthocyanins such as cyanidin-3-glucoside) [66,67].

### 2.3. Nitrogen-Containing Compounds 

#### 2.3.1. Alkaloids 

**Figure 3:**
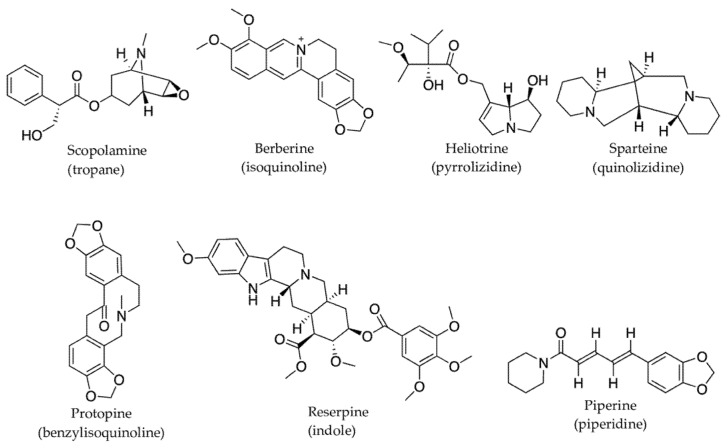
Representative examples of seven different types of alkaloids produced in plants and their chemical structure.

Alkaloids are the major group of plant defense molecules that contain a nitrogen atom(s) derived from the decarboxylation of amino acids and are known to occur in 20% of plant species [32]. There are seven types of alkaloids based on their amino acid precursors (Figure 3). Tropane, pyrrolidine, and pyrrolizidine alkaloids are derived from ornithine amino acid precursors; benzylisoquinoline from tyrosine amino acid precursors; indolequinoline from tryptophane amino acid precursors; and quinolizidine and piperidine alkaloids from lysine amino acid precursors [68]. Alkaloids are widely distributed among plant lineages and are particularly abundant in angiosperms. Individual plant species may contain fewer than five to more than 30 alkaloids (e.g., 74 alkaloids in *Catharanthus roseus*, 54 in *Strychnos toxifera*, and 39 in *Rauwolfia serpentina*) [68,69]. Generally, a plant family produces only one type of alkaloid, although a few families such as Solanaceae and Rutaceae accumulate a broad spectrum of alkaloids [70]. For example, *Duboisia myoporoides* R.Br. contains both a tropane alkaloid (hyoscine) and a pyridine alkaloid (nicotine) [71]. More than 20,000 alkaloids have been isolated, of which about 600 are known to be bioactive [72], but the exact physiological or metabolic role of alkaloids in plants remains poorly understood [68]. Alkaloids are best known for their defensive role as insect-herbivore deterrents owing to their characteristic bitter taste [73]. Thus, according to Levin [69], most alkaloid-bearing plants are found in the tropics, where intensive herbivore pressure is present. Defensive or toxic alkaloids in plants may be produced either by the plants themselves or by their symbiotic partners [74,75]. For example, the symbiotic endophyte *Epichloe coenophiala* in tall fescue grass [*Lolium arundinaceum* (Schreb.) Darbysh, syn. *Festuca arundinacea* (Schreb.), and *Schedonorus arundinaceus* (Schreb.) Dumort.) produces insecticidal alkaloids, lolines, and ergot, which cause ‘fescue toxicosis’ in grazing animals [76]. Alkaloid biosynthesis in plants is genetically controlled, but environmental factors such as light (UV), temperature, moisture, and soil nutrients also influence the type and rate of alkaloid production [76,77].

#### 2.3.2. Cyanogenic Glycosides and Glucosinolates

**Figure 4:**
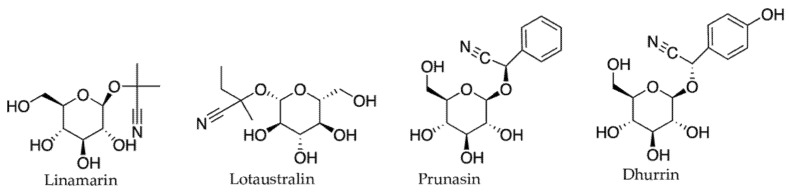
Examples of widely distributed cyanogenic glycosides in plant kingdom.

Other N-containing defense compound groups include cyanogenic glycosides and glucosinolates. These two groups are also derived from amino acid precursors and are significantly less diverse in their structure, with over a hundred compounds known from each group. Cyanogenic glycosides are reported in more than 2500 plant species [78], including ferns, gymnosperms, and angiosperms, while glucosinolates have been reported only in the order Capparales and in the genus *Drypetes* of the Euphorbiaceae [79]. According to Vetter [78] and Gleadow and Moller [80], some of the widely distributed cyanogenic glycosides in the plant kingdom are linamarin and lotaustralin (in Compositae, Linaceae, Fabaceae, Papaveraceae, and Euphorbiaceae); prunasin (in Myrtaceae, Polypodiaceae, Rosaceae, Saxifragaceae, Scrophulariaceae, and Myoporaceae); and dhurrin (in Poaceae and Euphorbiaceae) (Figure 4). Important food crops such as apple (*Malus domestica*), apricot (*Prunus armeniaca*), bamboo (*Bambusa vulgaris*), cassava (*Manihot esculenta*), cocoyam (*Colocasia esculenta* and *Xanthosoma sagittifolium*), and sorghum (*Sorghum bicolor*) are known to contain cyanogenic glycosides [81,82]. Cyanogenic glycosides and glucosinolates are generally higher in young leaves [83,84] and reproductive tissues [23,83,84,85]. They are toxic in higher concentrations [86], but in response to the low light, some plants such as tropical *Prunus turneriana* tend to accumulate more cyanogenic glycosides in older leaves. Although cyanogenic glycosides and glucosinolates in plants also respond to climatic stress such as drought and increased temperatures [80,86], they are not discussed in the following sections of this review.

## 3. Factors Influencing PSMs Production in Plants

**Figure 5:**
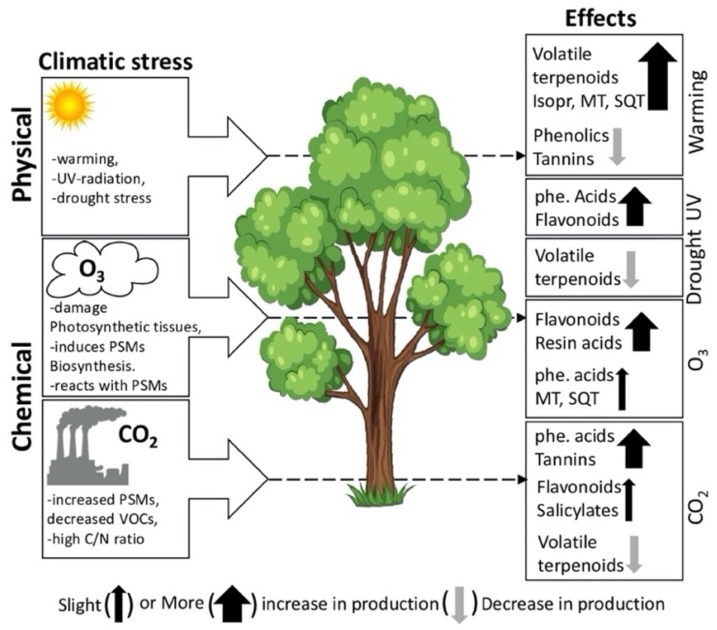
Abiotic stresses and their influence on the types of secondary metabolites in plants (adapted from [94,95,96,97]). Abbreviations: UV radiation = ultraviolet radiation; PSMs = plant secondary metabolites; O_3_ = ozone; CO_2_ = carbon dioxide; Isopr = isoprenoids; MT = monoterpenes; SQT = sesquiterpenes; phe. acids = phenolic acids.

Vickers et al. [87] have proposed two hypothetical mechanisms by which plants may respond to multiple external stressors: membrane stabilization and direct antioxidative scavenging of reactive oxygen species (ROS) generated under stressful conditions and to attract pollinators [88]. Under oxidative stress, plants either directly catalyze ROS to less harmful compounds using enzymes such as superoxide dismutase, catalase, and peroxidase or mediate enzymatic regeneration of antioxidants (e.g., monodehydroascorbate reductase, dehydroascorbate reductase, and glutathione reductase) [64]. Non-volatile isoprenoids such as tocopherols, zeaxanthin, and carnosic acid can scavenge ROS directly by reactions through hydroxyl radicals [89,90]. Interestingly, rising global temperatures and other environmental variables such as atmospheric O_3_ concentration and UV-B radiation are known to increase plant stress and, therefore, enhance or limit PSMs production as means to cope with such stressors. VOC emissions from plants are triggered by wounding and tri-trophic interactions (plant-herbivorous-carnivorous arthropods) [91] and they are influenced by various environmental factors, including temperature, light, moisture, and pollutants [92]. Individual stress has a selective influence on PSMs production, either by inducing or inhibiting the compound biosynthesis or emission based on stress conditions in plants (Figure 5). While PSMs have diverse functions in plants, their production also depends on multiple factors [34,93]. The effects of abiotic stress on PSMs production are given in Table 1.

### 3.1. Effects of Heat Stress on PSMs

Warming causes the accumulation of terpenoids, which usually have protective functions in mitigating environment-induced oxidative stress in plants [87,190]. For instance, tomato (*S. lycopersicum*) grown under heat stress (at 46 °C) emits higher levels of monoterpenes such as α-thujene, α-pinene, camphene, 2-carene, α-phellandrene, δ-3-carene (car-3-ene), α-terpinene, limonene, β-phellandrene, (*E*)-β-ocimene, and terpinolene; and also sesquiterpenes such as δ-elemene, β-elemene, α-humulene, and β-caryophyllene (Table 1) compared to controls [97]. In contrast, Nogués et al. [191] observed decreased emission of terpenes in *Citrus monspeliensis* grown under laboratory conditions at 35 °C; instead, increased assimilation of water-soluble antioxidant ascorbate indicates a shift from terpene-mediated to ascorbate-mediated ROS scavenging mechanism. Moreover, when *C. monspeliensis* was grown in the field, total terpene emission was higher during winter than in summer [191]. These contrasting findings suggest that terpene emissions under heat conditions could be species-specific and vary seasonally. Additionally, free fatty acids released by membrane phospholipase in response to heat (and cold) form lipoxygenase (LOX) products via lipoxygenase pathway, out of which C6 compounds (*Z*)-3-hexenal and (*E*)-2-hexenal are most common [97]. Wounded plants also release these two compounds within a few minutes [192,193]. Notably, (*E*)-2-hexenal acts as a chemical signal inducing the expression of stress-related transcription factors such as HSFA2 (heat stress transcription factor A-2) and MBF1c (multiprotein-bridging factor 1c) [194]. Heat stress may cause the melting of cuticular lipids, thus increasing cuticular permeability [195], and extreme temperatures may rupture terpene-containing-glandular trichomes, releasing the contents into the air [97]. After exposure to cold and heat stresses, favorable pH conditions inside plastids favor increased terpene synthesis [97] (Figure 5). 

Under simulated environmental conditions, heat stress damages membranes (e.g., thylakoid membrane) and disintegrates membrane protein complexes (e.g., photosystem II) [196], consequently decreasing the rate of photosynthesis. Plants counteract such damage through sustained synthesis and emission of terpenes [87,197]. Korankye et al. [197] proposed that plants produce more terpenes under stressful conditions by diverting carbon to a non-mevalonate pathway, which otherwise could have been used in photosynthesis. Monoterpenes such as 1,8-cineole, α-terpinyl acetate, linalyl acetate, limonene, sabinene, myrcene, α-terpinen, β-ocimene, α-terpinolene, and γ-terpinene are most produced following decreased photosynthesis in plants [191,198]. Non-targeted PSMs profiling in tomatoes revealed higher concentrations of α-tocopherol and plastoquinone under 38 °C compared to lower temperatures (20 and 10 °C) [199]. Taken together with other studies [200,201], this suggests that these compounds function as electron carriers and facilitate photosynthesis in addition to their anti-oxidative functions. The photosynthetic rate also decreases under the increasing temperature as in *Pueraria lobata* [Willd.] Ohwi., and *Quercus* spp. when isoprene synthesis (non-mevalonate pathway) was inhibited with fosmidomycin [202]. They suggest that isoprene improves thermotolerance in plants and helps photosynthetic apparatus recover after experiencing heat shock (i.e., temperature > 40 °C). Studies [203,204] suggest that plants tolerant to sunlight-induced heat flecks, O_3_, and ROS produce more isoprene than non-tolerant species. However, not all plants seem to produce isoprenoid compounds, but it varies among different plant species. For instance, when grown at 30 °C, *Salix phylicifolia* L. emitted isoprene, whereas *Betula nana* L. and *Cassiope tetragona* (L) D.Don emitted monoterpenes such as (*Z*)-2-hexenal, hexenyl butyrate, hexenyl acetate, and 3-hexenyl-methyl butanoate [205]. Heat stress also enhances the production of water-soluble antioxidants (e.g., ascorbate and glutathione) as well as lipid-soluble antioxidants (e.g., tocopherols) that scavenge increasing ROS [206,207]. For example, *Lycopersicon esculentum* Mill. Var. Amalia, after receiving heat shock at 45 °C for three hours, has been shown to produce more ascorbate and glutathione than its wild thermotolerant type Nagcarlang control under the same conditions [207]. Heat stress also affects flavonoids production as sweet basil (*Ocimum basilicum* L.) responds to high temperatures by producing flavonoids [208].

### 3.2. Effects of Cold Stress on PSMs

Cold stress or low-temperature stress is either chilling (<20 °C) or freezing (<0 °C) temperature, and they adversely affect plants’ growth and development. Plants growing in sub-tropical and tropical areas are more sensitive to cold stress than temperate species [209]. Cold stress tolerance in plants is achieved through selective expression of stress-defensive genes, which is reviewed by Chinnusamy et al. [210]. For instance, Jeon et al. [106] investigated transcripts and metabolites in six-day-old tartary buckwheat (*Fagopyrum tartaricum*) after cold exposure (at 4 °C, for various periods), observing upregulation of phenylpropanoid biosynthetic transcripts and significant accumulation of anthocyanins and proanthocyanidins, both antioxidative (Table 1) [107]. When two varieties of grapevine *Vitis vinifera* L. (cold tolerant – Maerchal Foch, and cold-sensitive – Kiszmisz Luczistyj) were exposed to 10/7 °C day/night cycle for 14 h photoperiod at 180–200 μm/(m2s) irradiance, the cold-tolerant variety had higher total phenolic compound content when assessed using the Folin-Ciocalteu’s reagent [211]. Subsequently, when they tested the antioxidant capacities of leaf extracts from two varieties by DPPH (2,2-diphenyl-1-picrylhydrazyl) free radical scavenging assay, leaves from cold-tolerant varieties yielded better activity. 

Another exciting example of the role of PSMs in plants under cold stress is the medicinal plant, Indian ginseng (*Withania somnifera* L.), which is the primary source of biologically active withanolides. Mir et al. [108] studied the accumulation of withanolides in response to cold stress in two genotypes of *W. somnifera* (AGB002―wild genotype and AGB025―cultivated genotype). After subjecting these two genotypes to chilling temperature (4 °C, for a maximum of seven days), bioactive compounds such as withanolide A in the roots and withaferin A in leaves were detected in both genotypes, suggesting the involvement of withanolides in cold tolerance. Moreover, the wild genotype showed a higher accumulation of marker withanolides than the cultivated one, which could mean that plants may not produce relevant bioactive compounds when out of their natural habitat, which is discussed later.

Glucosylated terpenoids (e.g., some sesquiterpenes) are another group of PSM involved in cold stress tolerance. Zhao et al. [111] reported the accumulation of glucosylated sesquiterpene and nerolidol glucoside (i.e., catalyzed by plant glycosyltransferase, UGT91Q2) in tea plants (*Camellia sinensis*) in response to cold stress (freezing temperature, −5 °C, for 4 h). The accumulation of nerolidol glucoside was directly proportional to the expression level of UGT91Q2, indicating that cold stress induces glycosylation in tea. Moreover, the ROS-scavenging ability of nerolidol glucoside was significantly higher than nerolidol, thus increasing cold tolerance in tea.

### 3.3. Effects of Drought Stress on PSMs 

Climate change is expected to alter precipitation patterns and results in drought stress (water deficit) in some plants. Drought stress is considered major abiotic stress that impedes metabolism [212,213] and leads to changes in plants at the morphological, physiological, biochemical, metabolic, and transcriptional levels. ROS formation is one drought stress effect, which damages cellular components, including proteins, lipids, and nucleic acids [214,215]. Accumulation of flavonoids such as flavonols and anthocyanins is essential in protecting against abiotic stresses, including drought stress, but the mechanism of action is poorly understood [216]. For example, concentrations of antioxidant flavonols epigallocatechin gallate, epicatechin, and epicatechin gallate increase in the leaves of *Cistus clusii* under drought stress, reaching a maximum after 30 days of exposure [120,217]. However, the efficacy of photosystem II (PSII) and lipid peroxidation remained unchanged. Under drought stress, PSII in the cotton (*Gossypium hirsutum*) also remained unaffected [218]. Nakabayashi et al. [216,219] also obtained a similar result (increasing flavonols and anthocyanins) under drought stress in the aerial parts of *Arabidopsis thaliana* (wild type, Col-0) and confirmed that overaccumulation of flavonoids is key to drought tolerance. There was also a drastic increase in the concentrations of glycosides of kaempferol, quercetin, and cyanidin along with drought stress marker metabolites (proline, raffinose, and galactinol). Excessive accumulation of anthocyanins protects plants against drought stress [219], and anthocyanins are thought to be more robust antioxidants due to their higher level of hydroxylation [220]. A few other studies [221,222] have reported similar observations, i.e., increased accumulation of anthocyanins in plants under drought. Drought stress in *Amaranthus tricolor* genotype VA3 increased concentrations of at least 16 phenolic compounds, including six hydroxybenzoic acids, seven hydroxycinnamic acids, three flavonoids, and a new phenolic acid, trans-cinnamic acid (Table 1) [112]. In tea plants, fulvic acid is the primary driver of tolerance against drought stress by enhancing ascorbate and glutathione metabolism and promoting flavonoids biosynthesis [223]. More examples and patterns of biochemical changes induced by drought stress in plants are given in Table 1. 

### 3.4. Effects of Ultraviolet (UV) Radiation on PSMs

Plants respond to excessive ultraviolet radiation (UV) both morphologically and physiologically. UV radiation is known to trigger a wide range of responses in plant cells, mainly by UV-B (280–320 nm) and less by UV-A (315–400 nm). Plants’ response to UV stress depends on their perception, signal transduction mechanism, and influence of gene expression [224]. Other environmental factors also influence response to UV-B stress in plants as UV radiation indirectly damages the photosynthetic apparatus by generating ROS [225]. Thus, plants have developed a mechanism to protect against UV radiation and allow photosynthetically active radiation (PAR) to reach mesophyll and palisade tissues in order to enable photosynthesis. Synthesizing UV-absorbing flavonoids is one mechanism to mitigate photoinhibition and photooxidative damage by either reducing UV penetration or quenching ROS. Flavonoids can absorb radiation in the UV region of the spectrum; thus, these compounds are responsible for filtering UV light in plants [226]. Unlike other lights of different wavelengths, UV-B radiation can damage DNA and chloroplasts, particularly photosystem II (PSII) and modify or inhibit gene expression due to its high energy, and they are absorbed by a wide range of molecules [227]. When Stapleton and Walbot [226] investigated DNA damage in maize plants exposed to UV-C or UV-B radiation at a dose of 6000 J/m^2^, maize plants with flavonoids, primarily anthocyanins, suffered less DNA damage than maize plants deficient in flavonoids. Flavonoids with a catechol group in their B-ring skeleton (e.g., quercetin derivatives) are best known to protect photosynthetic tissues from such oxidative damage [228]. Moreover, exposure to excess UV-B radiation causes increased synthesis of stronger antioxidants such as dihydroxy B-ring-substituted flavonoids (e.g., quercetin and luteolin glycosides) (Figure 5) and less effective antioxidant flavonoids such as kaempferol or apigenin glycosides [229,230]. As a response to UV irradiation, the concentrations of quercetin flavonoids increase in *Brassica napus* [156] and *Fagopyrum esculentum* [163]. The concentration of antioxidative flavonoids increased in *Kalanchoe pinnata* when exposed to UV-B radiation compared to ordinary white light [231]. When Del Valle et al. [225] investigated the effects of UV radiation in *Silene littorea*, UV exposure increased the concentrations of protective phenolic compounds but affected its reproductive efficacy. UV-B radiation modifies gene expression, but their underlying molecular mechanism is not well understood, unlike other phytochrome and blue/or UV-A. Herrlich et al. [232] attribute plant response to UV-B stress mainly to damage caused to cell membranes and DNA. The multiple roles of flavonoids, including photoprotection and the effects of stress on flavonoid biosynthesis, are reviewed elsewhere [52,54]. 

### 3.5. Effects of Ozone on PSMs

Ozone (O_3_) in the lower atmosphere (troposphere) acts as a greenhouse gas and is phototoxic to plants [233]. It is usually produced by reactions between primary pollutants (such as carbon oxides, sulphur oxides, nitric oxides, and hydrocarbons) catalyzed by sunlight. Although O_3_ is neither a free radical nor a ROS, its strong oxidizing properties enable it to react with biomacromolecules, including lipids, proteins, nucleic acids, and carbohydrates [234]. Generally, O_3_ enters through stomata and damages leaf tissues, mainly in the upper (adaxial) layers resulting in chlorosis and lesions. Physiologically, exposure to O_3_ impairs stomatal function (dysfunction of transpiration and water use efficiency) and reproductive development, CO_2_ assimilation, and subsequently photosynthetic activity. In snap bean (*Phaseolus vulgaris*), exposure to an ambient concentration of O_3_ (≤150 ppb, 1 h) [along with water stress (≤15%)] induces sluggishness in stomatal closure, subsequently causing more significant loss of leaf surface water [235]. 

**Figure 6:**
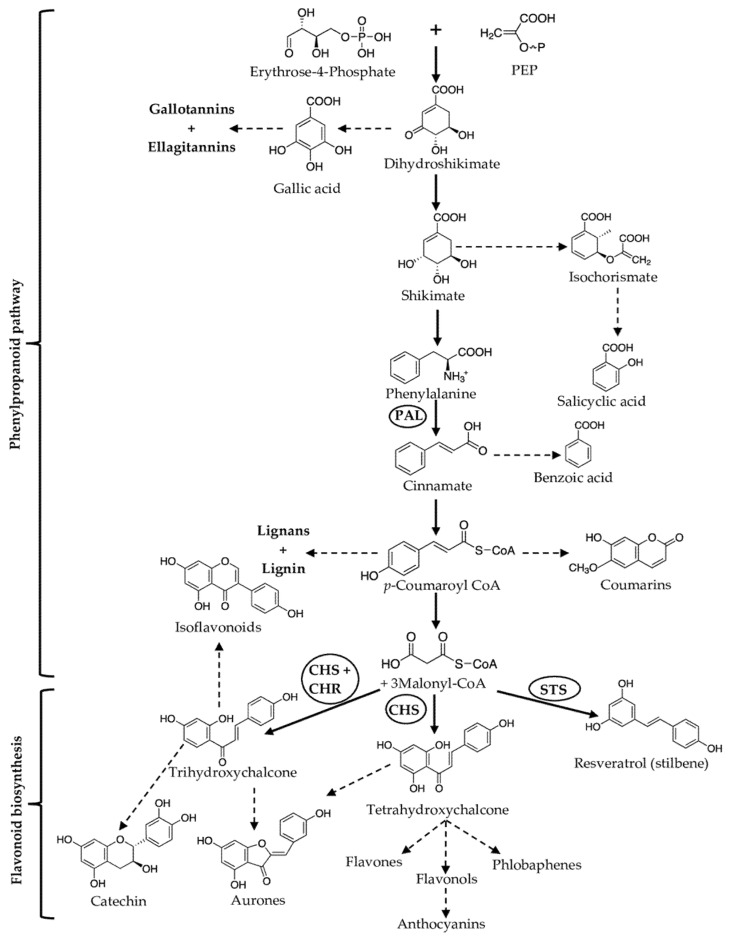
General phenylpropanoid pathway and flavonoid biosynthesis (adapted from [247,248]. Solid arrows represent single enzymatic reaction; dashed arrows represent multiple sequential reactions. Enzymes involved: PAL—phenylalanine ammonia lyase; CHS—chalcone synthase; STS—stilbene synthase; CHR—chalcone reductase.

In addition to changes in plant physiological functions, O_3_ triggers pathways responsible for producing defensive molecules, such as flavonoids. When Mao et al. exposed soybean leaves to elevated O_3_ (110 ± 10 nmol mol^−1^ for 8 h daily, for 54 days), the concentrations of rutin, quercetin, and total flavonoids increased significantly [236]. Ozone also enhances the activity of enzymes involved in flavonoid biosynthesis. Plants fumigated with O_3_ show increased activities of phenylalanine-ammonium lyase (PAL), and chalcone synthase (CHS) enzymes involved in phenylpropanoid and flavonoid biosynthesis pathways [237] and subsequently produce protective compounds that can scavenge ROS [56]. The general phenylpropanoid pathway and flavonoid biosynthesis pathways are outlined in Figure 6 below. These pathways, in turn, contribute significantly towards plant defense response by producing protective phenolic compounds such as condensed tannins and flavonoids that can scavenge ROS [57]. For instance, when Arabidopsis thaliana is exposed to O_3_ (300 ppb daily for 6 h), PAL mRNA levels increase 3-fold compared to their control plants [238]. Similarly, O_3_ treatment (200 nL/L for 10 h) increases both PAL and CHS activities resulting in a 2-fold increase of total leaf furanocoumarins and flavone glycosides in parsley (*Petroselinum crispum*) [239]. Lignin deposition in O_3_ exposed leaves is also linked to increased PAL activity [240], whereas in sage (*Salvia officinalis*), both PAL and PPO (phenol oxidase) activities were suppressed after 24 h exposure to O_3_ [241]. However, rosmarinic acid synthase (RAS) activity is accompanied by the increased transcription level of genes (e.g., RAS) encoding biosynthesis enzymes, suggesting that the sage plant mediates oxidative damage through synthesizing phenolic compounds. 

Studies have shown that plant chemical responses to O_3_ exposure variably depend on the O_3_ concentration [242]. Ozone alone enhances the production of phenolic compounds more significantly than in response to the increased CO_2_ concentration, while the combination of these two factors resulted in higher diterpenes, but not mono- and sesquiterpene, synthesis in plants [243]. However, some experiments showed contrasting results from O_3_ fumigation. Leaves of *Ginkgo biloba*, upon fumigation with an elevated level of O_3_, increased the concentrations of terpenes (Table 1), but phenolics decreased [152]. Ozone also enhances the accumulation of salicylic acid (SA) in plant tissues; for instance, in the tobacco plant (Nicotiana tabacum), emission of SA-derived methyl salicylate increases upon exposure to O_3_ [244,245]. In *Arabidopsis*, SA accumulation is necessary for forming O3-induced mRNAs, such as PAL and pathogenesis-related protein 1 (PAR1) transcripts [245]. Nevertheless, some plants (such as tobacco plants) do not require SA accumulation to form PAL transcripts [246]. These examples suggest that O_3_ induces at least two signaling pathways, the SA-dependent pathway associated with pathogen defense response and the SA-independent pathway in the protective response to O_3_.

Isoprene in tree foliage is known to protect foliage from oxidative stress. For instance, when Loreto et al. [204] applied isoprene (2–3 ppm) exogenously to tobacco and birch leaves fumigated with O_3_ (300 ppb), photosynthesis was consistent throughout the treatment period with the less accumulation of ROS compared to their fosmidomycin-treated control (showed more ROS accumulation and decreased rate of photosynthesis). Moreover, after three days of O_3_ treatment, they observed that areas of leaves treated with isoprene were intact, suggesting that isoprene protects photosynthetic tissues and stabilizes the thylakoid membrane. Isoprene protects photosynthesis in those plants exposed to acute thermal and O_3_ stress through antioxidative action (quench H_2_O_2_) and preventing membrane lipid peroxidation. For instance, leaves of *Phragmites australis* for which their endogenous isoprene production was inhibited by applying fosmidomycin become more sensitive to O_3_ stress than isoprene-producing leaves [204].

Exposure to high O_3_ concentration causes VOC emission, but at chronic O_3_ level, it modifies compositions of BVOCs, consequently affecting tri-trophic interactions and weakening plants’ response to arthropod attack [245,249]. During such situations, isoprenoids (mainly hemiterpenes, monoterpenes, and sesquiterpenes) are synthesized by plants to tolerate O_3_-induced damages. Hemiterpene is an example of an isoprenoid released in the leaves, as it can protect photosynthetic apparatus and scavenge O_3_ by-products and ROS due to its antioxidative activity [204]. The effect of O_3_ on alkaloid biosynthesis remains less elucidated, but polyamines in plants, which is an important alkaloid precursor, are correlated to O_3_ tolerance [234]. Polyamines in plants possess a wide array of physiological functions [240] in addition their involvement in response to both abiotic and biotic stresses [250].

## 4. Reported Pharmacological Properties of PSMs Present in Plants Affected by Ex Situ Abiotic Stresses

**Figure 7:**
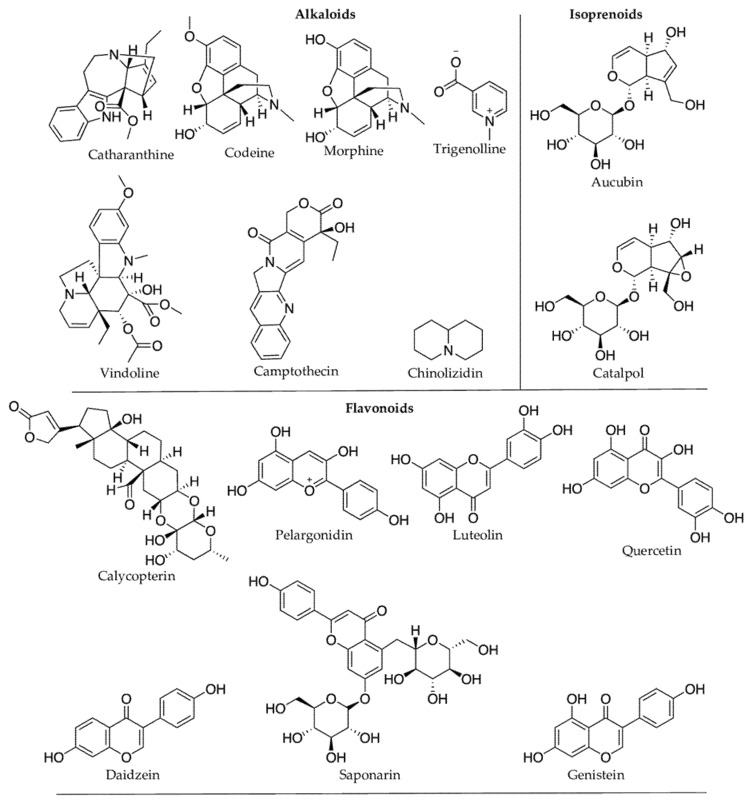

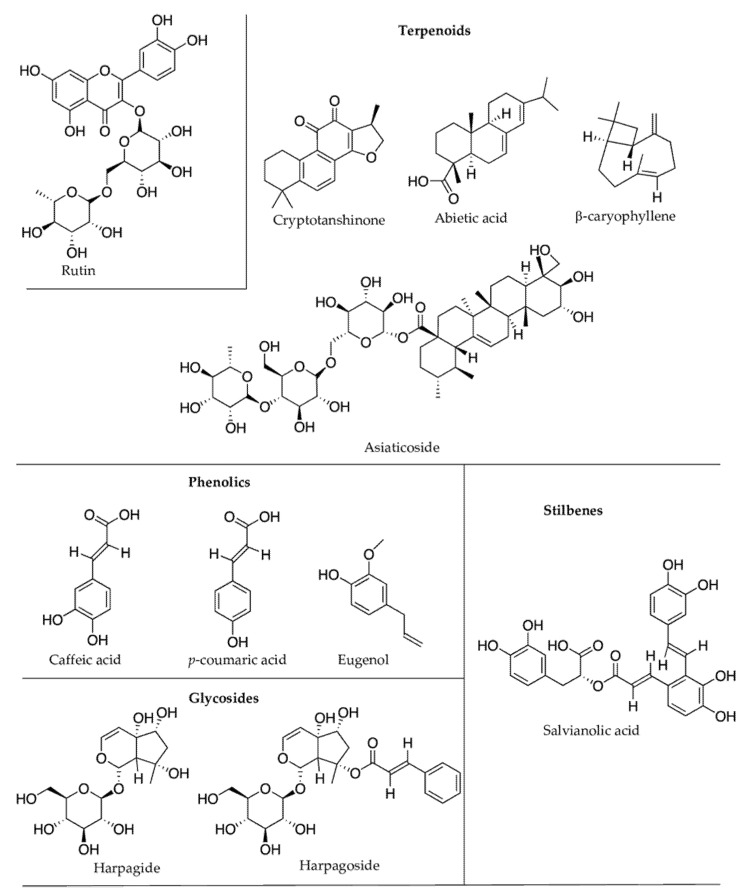
Chemical structure of compounds known to accumulate in plants under various abiotic stress conditions.

Plant protective secondary metabolites are diverse in structure and biological properties, and they have been continuously exploited for pharmaceutical, nutraceutical, and cosmetic uses [251] (Figure 7). Flavonoids and other phenolic compounds are predominant among secondary metabolites produced in response to climatic/or abiotic stress (Table 1). Flavonoids confer protection against inflammation, allergy, and bacterial infections [252]. Flavonols (or 3-hydroxy flavones), one of the main subclass of flavonoids, are apparent antioxidants in stressed plants, and they are known to prevent nuclear DNA damage by free radicals like H_2_O_2_ [253]. Flavonols are polyaromatic secondary metabolites with three rings, and many of them are bioactive. Many flavonoids possess antiviral properties. For instance, the hydroxy (OH) group in the ring-C of flavonols makes them more effective against herpes simplex virus type I than flavones [254]. Fisetin is another example of an active flavonoid produced by plants under oxidative stress, preventing membrane lipid peroxidation, DNA damage, and protein carbonylation [247]. Fisetin showed numerous biological activities such as protection against cell death from oxidative stress, growth, and maintenance of nerve cells (primary cortical neurons from a rat) [248,255]. Fisetin suppresses many inflammatory pathways, including Nuclear Factor-kappa B (NF-kB) pathway, helping prevent cancerous growth [256,257]. Similarly, Hussain et al. [258] also observed the protective effect of fisetin against smoke-induced oxidative stress and inflammation in rat lungs. Plant UV filters, kaempferol, and quercetin are a few other examples of bioactive flavonoids. Kaempferol is an anti-inflammatory [259], chemo-protective [260], and cardio-protective [261]. Polyphenolic resveratrol is one of the essential stilbene phytoalexin produced by a plant’s defense mechanism, and it possesses antioxidant, anticancer, and anti-estrogenic properties [262]. The immunoinhibitory compound, calycopterin isolated from the medicinal plant *Dracocephalum kotschyi* [168], was elevated upon UV irradiation in *Gnaphalium luteo-album* [167]. Tanshinones are other examples of bioactive phenols. In response to severe drought stress, their concentration in the *Salvia miltiorrhiza* increases, including tanshinone I and tanshinone IIA by 182% and 322%, respectively, compared to 148% under the moderate drought stress [139]. Tanshinones are known for their anti-inflammatory, antioxidant, and anticancer properties [263].

Nitrogen-containing compounds, alkaloids, are another group of secondary metabolites widely produced in plants for defense, and they are known to exhibit diverse biological activities, including anti-inflammatory, anti-malarial, and anticancer activities [264]. The fungistatic activity of α-tomatine (*Solanum* and *Lycopersicon* species) in *Fusarium oxysporum* f. *lycopersici* (tomato wilt) was the first bioactive alkaloid reported in 1945 by Irving et al. [69]. Alkaloids and their precursors accumulate more in plants when exposed to various stress factors. For example, *Catharanthus roseus*, when exposed to UV-B radiation, synthesizes more indole alkaloids and precursors of vinblastine and vincristine increase in hairy roots [265]. These alkaloids inhibit cell mitosis by destroying microtubules of the mitotic apparatus, blocking cancer cell division [266]. Bioactive alkaloids accumulate in response to high temperature, drought, and UV-B stresses (Table 1). Indole alkaloid vindoline from *Catharanthus roseus* (which increases in response to UV-B) showed anti-diabetic (reduces fasting blood glucose level) and anti-inflammatory (reduces pro-inflammatory cytokines, TNF- α and IL-6) properties [99]. 

The number of structurally determined specialized plant terpenes exceeds 105, including >12,000 diterpenoids [267]. Plant terpenoids are diverse and have been a valuable source of medicinal discoveries because terpenoids are natural NF-kB signaling inhibitors with anti-inflammatory and anti-cancer properties [268]. Examples include monoterpenes (e.g., (−)-menthol and cannabinoids); sesquiterpenes (e.g., artemisinin and thapsigargin); diterpenes (e.g., paclitaxel and ingenol mebutate) and triterpenes found in floral and vegetative parts; triterpenoids; and carotenoids (e.g., steroidal alkaloids, cardenolides, and bixin) (Figure 7). Other compounds are partially derived from a terpene precursor, such as monoterpenoid alkaloids (e.g., strychnine), which are synthesized in part from secologanin (Figure 7), a member of the widespread class of iridoid monoterpenes [269]. 

## 5. Biodiscovery Potential of Plants Growing under Ex-Situ Abiotic Stresses

Natural products, including PSMs, have been a significant source of medicines. According to Newman and Cragg, between 1981 and 2010, 1073 small molecules (mol. wt. < 1000 Da) were approved as new chemical entities, out of which more than half were from natural products [270]. An additional 321 small molecules were reported in another review published in September 2019 [271]. According to Butler et al. [272], in their review covering natural products-derived drugs between 2008–2013, 25 drugs were launched since 2008, and additional 31 compounds were in the last stage clinical trial (phase III). According to the database on www.clinicaltrials.gov (accessed 5 September 2021), four compounds have advanced to phase-IV clinical trial, sixteen have completed phase-III, nine have not yet completed phase-III, and two compounds have been withdrawn. The four compounds that have advanced to clinical trial phase-IV are oritavancin (anti-bacterial), ipragliflozin, tofoglifozin (anti-diabetic, type II diabetes), and vorapaxar (anti-thrombotic) [272]. Recently, pharmaceutical industries and researchers have renewed their interest in PSMs due to advancements in cutting-edge technology, including various chromatography and high-resolution spectroscopy tools and omics platforms [273].

Interestingly, not many PSMs were subjected to clinical trials. The reasons are varied. One of the continuing challenges for drug discovery from plant sources is obtaining enough sample extracts and compounds for testing in vitro and in vivo disease models. This bottleneck is heightened for species in the IUCN red list of threatened or endangered species prohibited for large sample collection, even if they show biological hits. While cultivating pharmaceutically interesting plant species may be a solution, it is not always possible to culture the organism outside its natural habitat. Even when possible, relevant natural products may not be produced outside their natural habitat [273]. Alternatively, plants affected by climate change could be a potential source of novel drug leads, considering the vast diversity of phytochemicals produced by them in response to various abiotic stress conditions (Table 1). 

Climate change rapidly and severely affects plant ecosystems; for instance, mountaintop ecosystems are sensitive to small shifts in temperature and precipitation patterns [274]. Several studies on the mountaintops of the Asia-Pacific region [275], Oceania [276], and Europe [277] have reported accelerated plant ecological responses, including distribution, ecophysiology, and interaction with other organisms due to climatic changes. In overcoming climate change-induced/or abiotic stress and finding an optimal climate niche, plants produce diverse PSMs, which could be of pharmaceutical interest. For example, the synthesis of plant terpenoids increases under heat, cold, and O_3_ stress, and the yield of many biologically active compounds also increases in plants grown in simulated environments of various abiotic stress conditions (Table 1). Abiotic stresses elicit bioactive compound synthesis [278], such as phenylpropanoids biosynthesis (mainly through shikimate pathway), causing an accumulation of compounds with defense or signaling functions (e.g., phenolics, flavonoids, and alkaloids) [279]. Similarly, it is reported that drought stress increases the concentration of camptothecin (anticancer alkaloid) in *Camptotheca acuminata* [116,117] and morphine (analgesic) concentrations in *Papaver somniferum*. The increased accumulation of PSMs in response to stress indicates that there may be novel bioactive alkaloid(s) in climate change-affected plants awaiting discovery. Abiotic stress factors under conditioned environment can potentially improve the yield of bioactive compounds in plants. 

## 6. Conclusions 

Plants constantly interact with the environment, and climate change has already impacted their diversity, growth, and survival. In order to minimize the impact of various climate change-related stresses (such as warming due to increased greenhouse gas emission, drought, cold, ozone-layer depletion, and harmful UV-radiation), plants produce diverse defense secondary metabolites, mainly phenolic and nitrogen-containing compounds. The biosynthesis of defense compounds in plants (including medicinal plants) is often upregulated, and these compounds are associated with various pharmacological properties, suggesting that plants affected by climate change may be a rich resource for drug discovery. However, most of these studies were conducted in simulated/or artificial environments. Thus, it would be interesting if more such studies (defense compounds produced by plants in response to climatic stress and their bioactivity) could be conducted by using plant samples from their natural habitats that are already challenged by the various climatic stresses. 

It is difficult to access various natural products bound by legislation and societal restrictions, including plants, for drug discovery research, particularly plants associated with indigenous knowledge. This limitation remains a considerable challenge for those working with medicinal plants. Other wild plants exposed to various climatic/or abiotic stresses would be an alternative option for drug discovery researchers. Another obstacle in the drug discovery process is obtaining adequate compounds for further biological tests (both in vitro and in vivo). Bioactive compounds increase their concentration in plants exposed to stress, for example, withanolides in Indian ginseng (*Withania somnifera*) increases in response to cold stress. Culturing plant tissues of interest at a large scale under a conditioned environment using various abiotic stresses can potentially improve the yield of bioactive compounds from plants. Thus, plant tissue culture would be another platform for researchers and pharmaceutical industries to upscale the production of valuable phytochemicals under duress of climate change factors.

## Figures and Tables

**Table 1:** Plant secondary metabolites produced in response to abiotic stresses and their reported pharmacological properties.

Stress Condition(s)	Plant Species (Family)	PSMs Produced	Effects on PSMs Concentration	Compound Class	Bioactive Compounds	Reported Pharmacological Properties
Cold stress	*Catharanthus roseus* (Apocynaceae) [98]	vindoline	Decrease	Alkaloids	vindoline	Antidiabetic [99]
Cold stress	*Glycine max* (Fabaceae) [94]	genistein, daidzein	Increase	Phenolics	genistein, daidzein	Antiproliferative [95,96]
Cold stress	*Solanum lycopersicon* (Solanaceae) [87,97]	(*Z*)-3-hexenol and (*E*)-2-hexenal (dominant); 1-hexanol and 1,4-hexadienal (smaller quantities)	Increase	Fatty Acyls	(*E*)-2-hexenal	Antibacterial [100]
Cold stress		β-phellandrene, (*E*)-β-ocimene	Increase	Terpenoids	NA	NA
Cold stress		δ-elemene, α-humulene and β-caryophyllene (dominant); in severe cold: β-elemene is produced.	Increase	Terpenoids	δ-elemene, α-humulene and β-caryophyllene	Antiproliferative [101]; anticancer [102]; anti-inflammatory [103]
Cold stress	*Zea mays* (Poaceae) [104]	pelargonidin	Increase	Phenolics	pelargonidin	Antithrombotic [105]
Cold stress	*Fagopyrum tartaricum* (Polygonaceae) [106]	anthocyanins (e.g.,3-*O*-galactosides) and anthocyanidins (e.g., malvidin)	Increase	Phenolics	anthocyanins	Antioxidant [107]
Cold stress	*Withania somnifera* (Solanaceae) [108]	withanolide A, withaferin A	Increase	Terpenoids	withanolide A; withferin A	Neuroprotective [109]; anticancer [110]
Cold stress	*Camellia sinensis* (Theaceae) [111]	nerolidol glucoside	Increase	Terpenoids	NA	NA
Drought	*Amaranthus tricolor* (Amaranthaceae) [112]	hydroxybenzoic acids (gallic acid, vanillic acid, syringic acid, *p*-hydroxybenzoic acid, salicylic acid, ellagic acid), hydroxycinnamic acids (caffeic acid, chlorogenic acid, *p*-coumaric acid, ferulic acid, *m*-coumaric acid, sinapic acid, *trans*-cinnamic acid), flavonoids (iso-quercetin, hyperoside, rutin).	Increase	Phenolics (Flavonoids)	*p*-hydroxybenzoic acid	Antisickling activity [113]
Drought	*Camellia sinensis* (Theaceae) [114]	Epicatechins	Increase	Phenolics (Flavonoids)	epicatechins	Antioxidant [115]
Drought	*Camptotheca acuminata* (Nyssaceae) [116]	camptothecin	Increase	Alkaloids	camptothecin	Antitumour [117]
Drought (PEG-induced)	*Catharanthus roseus* (Apocyanaceae) [118]	vinblastine	Increase	Alkaloids	vinblastine	Anticancer [119]
Drought	*Cistus clusii* (Cistaceae) [120]	epigallocatechin gallate, epicatechin, epicatechin gallate, and ascorbic acid.	Increase	Phenolics (Flavonols)	epigallocatechin gallate	Anticancer [121]; antibacterial [122]
Drought	*Crataegus laevigata*, *C. monogyna* (Rosaceae) [123]	chlorogenic acid, catechin, (−)-epicatechin	Increase	Phenolics	chlorogenic acid, (−)-epicatechin	Antioxidant [124,125]
Drought	*Glycine max* (Fabaceae) [126]	trigonelline	Increase	Alkaloids	trigonelline	Antidiabetic [127]
Drought	*Hypericum brasiliense* (Hypericaceae) [128]	isouliginosin B, rutin, 1,5-dihydroxyxanthone	Increase	Phenolics	isouliginosin B, rutin,	Antinociceptive [129]; Anticancer [130]
		betulinic acid		Terpenoids	betulinic acid	Anticancer [131]
Drought	*Lupinus angustifolius* (Fabaceae) [132]	chinolizidin	Increase	Alkaloids	NA	NA
Drought	*Papaver somniferum* (Papaveraceae) [133]	morphine, codeine	Increase	Alkaloids	morphine, codeine	Analgesic [134,135]
Drought	*Pinus sylvestris* (Pinaceae) [136]	abietic acid	Increase	Terpenoids	abietic acid	Antiallergic [137]; anti-inflammatory [138]
Drought	*Salvia miltiorrhiza* (Lamiaceae) [139]	tanshinones, cryptotanshinone	Increase	Terpenoids	cryptotanshinone	Anticancer [140].
Drought	*S. miltiorrhiza* [139]	rosmarinic acid	Decrease	Phenolics	rosmarinic acid	Antioxidant [141]
		salvianolic acid	Increase		salvianolic acids	Antioxidant [142]
Drought	*Scrophularia ningpoensis* (Scrophulariaceae) [143]	catalpol, harpagide, aucubin, harpagoside	Increase	Glycosides	catalpol, aucubin	Hepatoprotective [144]; neuroprotective [145]
Ozone (O_3_) stress	*S. lycopersicon* [87,97]	α-carotene, β-carotene, violoxanthin	Increase	Terpenoids	β-carotene	Antioxidants [146]; anti-inflammatory [147]
		isoprene, α-pinene, β-pinene, myrcene, limonene, sabinene, (*E*)-β-ocimene, (*Z*)-β-ocimene, α-humulene, (*E*)-β-farnesene, (*E,E*)-α-farnesene, (*E*)-β-caryophyllene, δ-cadinene	Increase	Terpenoids	α-pinene; myrcene; limonene; α-humulene.	Anti-inflammatory [148]; anti-asthmatic [149]; antioxidant [150]; anti-inflammatory [151]
O_3_	*Gingko biloba* (Ginkgoaceae) [152]	ginkgolide A	Increase	Terpenoids	ginkgolide A	Neuroprotective [153]
Ultraviolet radiation-B (UV-B)	*Arabidopsis thaliana* (Brassicaceae) [154]	kaempferol 3-gentiobioside-7-rhamnoside; kaempferol 3,7-dirhamnoside.	Increase	Phenolics (Flavonoids)	NA	NA
UV-B	*Brassica napus* (Brassicaceae) [155]	quercetin 3-sophoroide-7-glucoside; quercetin 3-sinapyl sophoroside-7-glucoside	Increase	Phenolics (Flavonoids)	NA	NA
UV-B	*Brassica oleracea* (Brassicaceae) [156]	cyanidine glycosides; sinapyl alcohol	Increase	Phenolics (Flavoboids)	NA	NA
UV-B	*C. roseus* (Apocynaceae) [157,158]	catharanthine, vindoline	Increase	Alkaloids	catharanthine	Anticancer [159]
	*Clarkia breweri* (Onagraceae) [160]	eugenol, isoeugenol, methyleugenol, and isomethyleugenol	Increase	Phenolics	eugenol	Antifungal [161]; anti-inflammatory [162]
UV-B	*Fagopyrum esculentum* (Polygonaceae) [163]	rutin, quercetin, catechin	Increase	Phenolics	quercetin; catechin	Antioxidant [164]; anticancer and antioxidant [165,166]
UV-B	*Gnaphalium luteoalbum* (Asteraceae) [167]	calycopterin; 3’-methoxycalycopterin	Increase	Phenolics (Flavonoids)	calycopterin	Anticancer [168]
UV-B	*G. viravira* [169]	7-O-methyl araneol	Increase	Phenolics (Flavonoids)	NA	NA
UV-B	*Hordeum vulgare* (Poaceae) [170]	saponarin; luteolin	Increase	Phenolics (Flavonoids)	saponarin; luteolin	Antihypertensive [171]; antibacterial [172]
UV-B	*Marchantia polymorpha* (Marchantiaceae) [173]	luteolin 7-glucuronide; luteolin 3,4’-di-*p*-coumaryl-quercetin 3-glucoside.	Increase	Phenolics (Flavonoids)	NA	NA
UV-B	*Quercus ilex* (Fagaceae) [174]	acylated kaempferol glycosides	Increase	Phenolics (Flavonoids)	kaempferol	Anticancer [175]; anti-inflammatory [176]
Heat stress	*C. acuminata* [177]	10-hydroxycamptothecin	Increase	Alkaloids	10-hydroxycamptothecin	Anticancer [178]
Heat stress	*Daucus carota* (Apiaceae) [179,180,181]	α-terpinolene	Decrease	Terpenoids	α-terpinolene	Antioxidant and anticancer [182]
		α-caryophyllene, β-farnesene	Increase		NA	NA
		anthocyanins, coumaric and caffeic acid;	Increase	Phenolics	*p*-coumaric acid and caffeic acid	Antioxidant [183,184]
Heat stress	*Q. rubra* (Fagaceae) [185]	isoprene (2-methyl-1,3-butadiene)	Increase	Terpenoids	NA	NA
Heat stress	*S. lycopersicon* [87,97]	β-phellandrene (dominant), 2-carene, α-phellandrene, limonene; increased emission of (*E*)-β-ocimene after treatment above 46 °C; β-caryophyllene.	Increase	Terpenoids	α-phellandrene; β-caryophyllene	Antifungal [186]; anticancer and anti-inflammatory [102,103]
		α-humulene	Decrease		α-humulene	Anticancer [187]
Heat stress (increased humidity)	*Centella asiatica* (Apiaceae) [188]	asiaticoside	Increase	Phenolics	asiaticoside	Anti-cellulite agent [189]

Abbreviations: NA: not available; LOX: lipoxygenase; UV: ultraviolet; ROS: reactive oxygen species.

## Data Availability

Not applicable.
